# Comparing molecular representations, e-nose signals, and other featurization, for learning to smell aroma molecules

**DOI:** 10.1371/journal.pone.0289881

**Published:** 2023-08-11

**Authors:** Tanoy Debnath, Samy Badreddine, Priyadarshini Kumari, Michael Spranger

**Affiliations:** Sony AI, SONY Corporation, Tokyo, Japan; Taipei Medical University, TAIWAN

## Abstract

Recent research has attempted to predict our perception of odorants using Machine Learning models. The featurization of the olfactory stimuli usually represents the odorants using molecular structure parameters, molecular fingerprints, mass spectra, or e-nose signals. However, the impact of the choice of featurization on predictive performance remains poorly reported in direct comparative studies. This paper experiments with different sensory features for several olfactory perception tasks. We investigate the multilabel classification of aroma molecules in odor descriptors. We investigate single-label classification not only in fine-grained odor descriptors (‘orange’, ‘waxy’, etc.), but also in odor descriptor groups. We created a database of odor vectors for 114 aroma molecules to conduct our experiments using a QCM (Quartz Crystal Microbalance) type smell sensor module (Aroma Coder®V2 Set). We compare these smell features with different baseline features to evaluate the cluster composition, considering the frequencies of the top odor descriptors carried by the aroma molecules. Experimental results suggest a statistically significant better performance of the QCM type smell sensor module compared with other baseline features with F1 evaluation metric.

## Introduction

Among our five senses, smell and taste are responsible for chemical perception and recognition. There are likely several hundred distinct odorants that humans can smell. Any possible mixture of these odorant molecules creates a new point in the odor space that may be completely different or almost identical to the original odor of the molecule [1]. Signals from activated olfactory neuron circuits are aggregated and transferred to the olfactory bulb, producing patterns that are processed at higher parts of the brain. Then, this olfactory information is encoded into perception and linguistic descriptors: ‘fruity’, ‘sweet’, ‘peach’, etc. Significant advances in vision, audition, and speech [2] suggest that it is possible to similarly predict the sensory output for olfactory stimuli. Progress in the olfactory domain could help us automatically discover new odorant molecules in a field where perfume and food industries mostly depend on manual methods.

However, the available databases for olfactory perception research remain limited, unlike other deep learning-based research domains such as computer vision. The output space generally consists of odor descriptors scores obtained from a sensory test. There are two types of sensory datasets available in the olfactory perception domain. One type represents sensory data using continuous values [3, 4], either via several intermediate levels or a range from, for example, 0 to 100 that describes the intensity of a given odor descriptor (‘sweet’). The second type is binary, that means, an odor descriptor is either detected or not detected for a given sample [5–7]. The continuous valued data (Dravnieks [3] & Dream dataset [4]) is typically small with a few hundred samples, whereas binary-valued data (Good scents [6], Leffingwell [5]) have larger sizes with up to a few thousand samples. Creating a large continuous value database is difficult because people evaluate odor impression scores on a full scale from 0, so it is a very time-consuming task. On the other hand, publicly available binary valued databases are much larger than continuous valued data because only odor descriptors (e.g., ’fruity’, ’floral’) are recorded for a flavor molecule.

Choosing a set of input features representing aroma molecules and other odorants remains an open challenge. One option is to generate thousands of physicochemical features for each molecule through the Dragon (closed source) [8] and Mordred (open source) [9] software. These consists of atom types, functional groups, topological indices, ring descriptors etc. We also often see the use of Simplified Molecular-Input Line-Entry System (SMILES) that can be calculated by Cheminformatic tool (RDKit) [10]. For a given SMILES representation, we can get the molecular fingerprints [11, 12] (Bit-based fingerprint) or count-based Morgan fingerprints (CFP).

Many works have approached odor impression prediction from these physicochemical properties. Sobel et al. [13] used PCA and linear models to explain the relationship between physicochemical parameters of odorants and odor properties. Keller & Vosshall [4] used 4885 molecular features for 476 molecules to establish a relationship between the odorants and their perceptual qualities. Following the above study, the DREAM olfaction prediction challenge demonstrated that it is possible to predict odor from molecular structures using regularized linear models [14]. Shang et al. [15] studied the method of predicting odor descriptors related to odor molecules using parameters of molecular structure. Hayashi et al. [16] used Pearson Correlation Coefficient maps (PCC- maps) and the t-distributed Stochastic Neighbor embedding (t-SNE) method to study the relationship between the odor maps and the molecular structure parameters of odorants. The Google research brain team used Graph Neural Networks with molecular structures to predict 138-odor descriptors tasks [17]. In summary, there have been many works for predicting odor impressions using molecular structure parameters. However, most of these works used publicly available datasets and only measured the prediction performance of one type of feature representation at once. Moreover, these methods cannot be used for chemical mixture even if their accuracy was good, as only simple molecules can be represented with these physicochemical features.

Another option to represent odors in features is mass spectrum sensory data. Mass spectrometry finds the fingerprint of a chemical using sensors based on electron ionization. Nozaki et al. [18] designed a predictive model based on deep neural network utilizing the mass spectra of mono-molecular chemicals. To alleviate for language bias when using linguistic odor descriptors, Nozaki et al. [19] also proposed a predictive model incorporating the language modeling method Word2vec [20, 21]. They converted the output space from binary descriptor values to continuous, aggregate values using embeddings for each descriptor. Following the above work, Tanoy et al. [22] predicted the odor descriptor groups using mass spectra for chemical mixtures such as essential oils.

Recently, much research is focused on developing new sensory data using e-noses, with a common type of sensors using the QCM technology. Their price and usability are usually advantageous compared with mass spectrum sensors, although sensor drift is said to happen after a certain period. Hanaki et al. [23] attempted to estimate the impression of blended fragrance using the Fuzzy Learning Vector Quantification (FLVQ) method. Recently, Guo et al. [24] studied mapping from QCM and metal oxide sensor responses to nine odor descriptors using convolutional LSTM (Long Short-Term Memory) model for 44 odorants.

**Fig 1:**
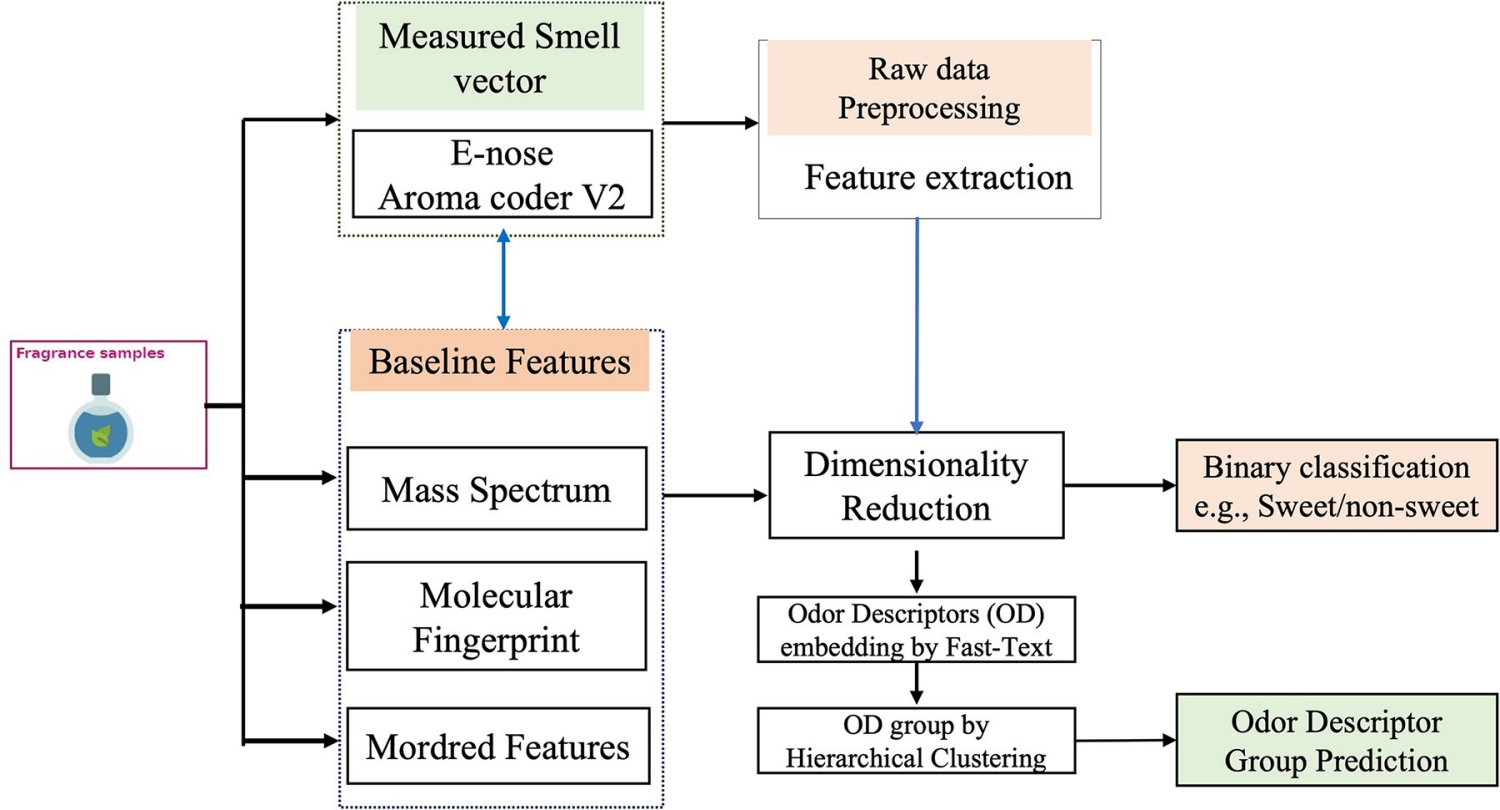
Workflow for modeling odor perception from different olfactory stimuli.

We found many different approaches in the literature to solve the prediction of odor impressions for a given odorant and its olfactory stimuli. Determining a featurization that can be used both for mono-molecular solutions and for chemical mixtures (which food and perfumes normally are) remains an ongoing challenge. However, to our knowledge, no study has evaluated the performance of prediction models in terms of different sensing and feature data in direct comparisons. Before reaching the long-term goal of building a model and system that predicts at best the odor perception from chemical mixtures, at first, we need to understand which input features is best used to represent odorants. In this study, we build a concrete understanding for modeling single odorant chemical using different representation of features illustrated in Fig 1. We gather molecular structure parameters, mass spectra, and molecular fingerprints for a common set of aroma molecules in the literature. We also build our own dataset of e-nose features using QCM type smell sensor module (Aroma Coder®V2 Set) and compare the prediction performance of individual odor descriptor & odor descriptor group with other baseline features.

## Materials and methods

### Data preparation

**Fig 2:**
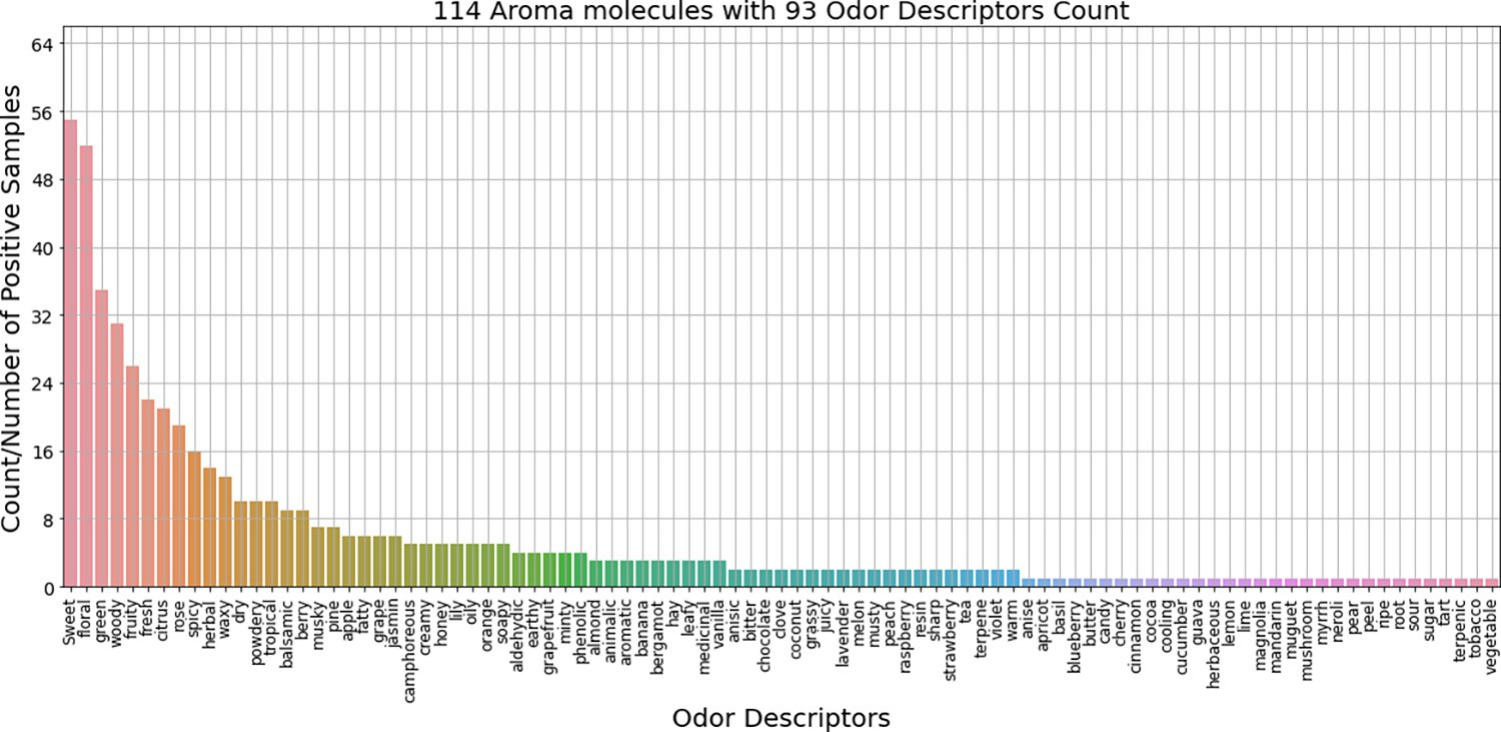
Odor Descriptors list with corresponding number of positive/target samples among 114 aroma molecules.

We focused on collecting a dataset of 114 molecules (after removing duplicate molecules) from fragrance kits (purchased [25] from http://profice.shop-pro.jp/)for experimenting with the Aroma Coder®V2 device, a QCM type sensor module. The manufacturer of the Aroma Bit device was not involved in choosing the odor molecules. We only purchased their device for measurement purposes. Therefore, there is no bias in the odor sample selection. Next, we collected odor descriptors (‘citrus’, ‘woody’, etc.) for each sample in the kit using the expert annotation Good Scents perfume materials database listed in S1 Data. Fig 2 depicts the list of odor descriptors with the corresponding numbers of samples carrying that descriptor among the 114 aroma molecules. We also joined the CAS number and SMILES for each molecule while collecting the dataset.

### Baseline features collection

In our baseline experiments, we compared the bit-based path descriptors fingerprints (bFP), the count-based Morgan fingerprints (cFP), the Mordred features, and the mass spectrum of each molecule, with the smell vector collected using the QCM-type smell sensor module.

The cheminformatic package RDKit was used to calculate the molecular fingerprint (bFP & cFP) with the following parameters: radius = 3, which gave us a 1024 bits number for a given SMILES string per molecule. Mordred features were also generated [9] using the RDKit software and we obtained 1324 features (these features are called molecular descriptors such as number of carbon atoms, molecular weight, predictive values of LogP and other molecular properties) for each molecule after removing the non-numerical features in the resulting parameters. Moreover, we collected the mass spectra of the aroma molecules from the chemistry Webbook [26] provided by National institute of Standards and Technology (NIST) using their corresponding CAS numbers where mass spectrum acquired with electron ionization of 70 [eV]. However, we were only able to obtain mass spectra for 80 of the 114 molecules and we included the exhaustive list of those 80 molecules & corresponding mass spectrum in S1 Data. As such, the experiments that compare mass spectra with the other features are conducted on a subset of 80 molecules in the dataset. Although we could not collect the mass spectra for all 114 molecules in this study, we still report the results on this reduced dataset because mass spectra are an important sensing data. Notably, as linear superposition is valid in mass spectrometry [27], it is the only baseline feature with the QCM-type sensor features that be used for both mono-molecule and chemical mixtures.

### Aroma bit smell vector measurement and feature extraction

**Fig 3:**
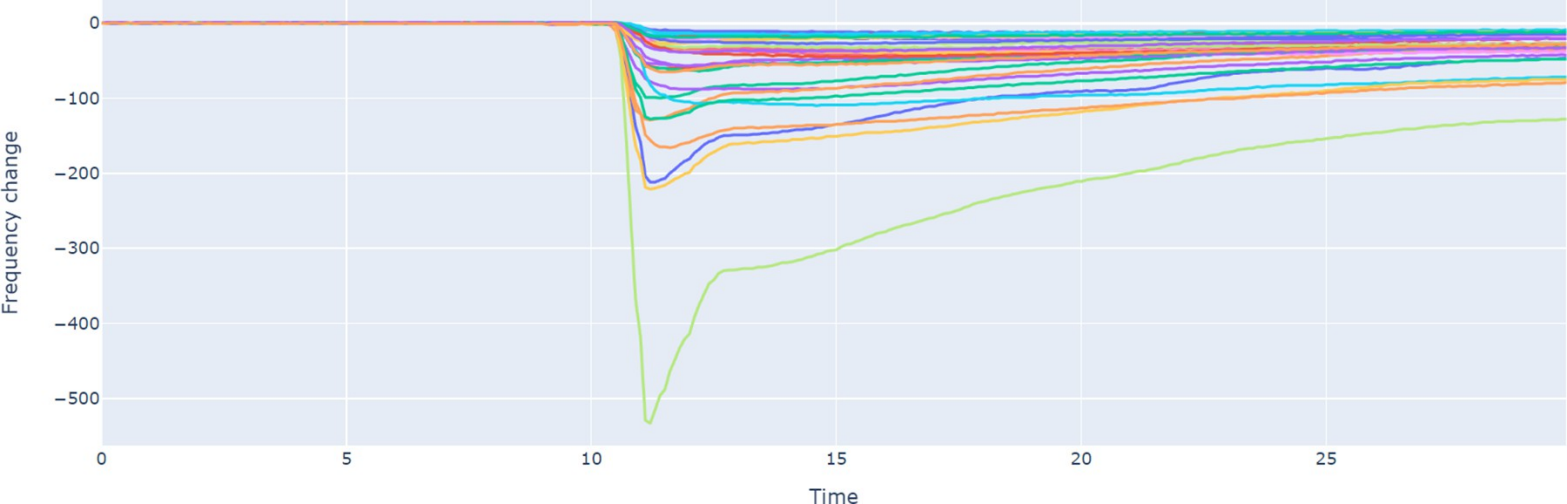
Aroma Bit output for the molecule ‘1,8-cineole’: Time vs Frequency change where each signal corresponds to one of the 35 different types of receptor membranes.

The Aroma Coder®V2 (ACV2) set is a desktop type odor measurement system developed by Aroma Bit, Inc., a start-up that develops compact odor sensors. It is equipped with 5 types of receptor membranes per module. ACV2 embeds 7 sensor modules, or 35 different types of receptor membranes per scan. These sensors convert the smell samples into electronic response data. Our measurement method of liquid aroma samples follows the instructions of the ACV2 set. We place 1 mL of a sample in a glass jar. The jar has a shutter lid that is connected to the air inlet of a detector unit. We let the sample rest for 2 minutes during which the smell fills the headspace of the jar. Then, we start the sensing procedure which lasts 30 sec. In the first 10 sec, the shutter opens, and the inlet of the detector unit pumps the air filled with smell from the jar. The sensor returns smell data as time series representing the frequency change over time of each receptor membrane. It takes a reading every 0.1sec. Thus, we obtain 35*300 features per sample. We convert these readings into 35-D vectors by taking the total difference between the base frequency reading (before opening of the shutter) and the peak frequency reading in these 300 intervals as shown in Fig 3. This is calculated independently for each of the 35 membranes. For each aroma vector, we sample three measurements using the afore-mentioned procedure and averaged their 35-dimensional vectors to obtain our set of features. We provided 35-dimensional average smell vectors in S1 Data.

### Predictive model & evaluation metric

Odor Prediction tasks are often considered as imbalanced problems [28]. With an increase in the specificity of the odor descriptors, the number of samples in each target class decreases. Fig 2 demonstrates this as most descriptors are present in less than a dozen samples. At first, we utilized a typical binary classifier (binary SVM) for predicting odor descriptors, but its performance on the imbalanced data was unsatisfactory as already reported before [28]. Out of several techniques to solve such imbalanced problems, we chose an algorithm level solution by using one class support vector machine (OCSVM). Schölkopf et al. [29] separates all data points from the origin and maximizes the distance from this hyperplane to the origin. This creates a binary function that captures regions of the input space. The objective function of one class support vector machine is slightly different (C parameter is used for binary SVM and ν parameter is used for One class SVM) from the typical SVM one.


$${min}_{w,b,\mathrm{\xi i}}\frac{{\left|w\right|}^{2}}{2}+\frac{1}{\nu n}\sum_{i=1}^{n}\mathrm{\xi i}-b$$

Subject to:

$$w.\varphi (x_{i})\geq b-\mathrm{\xi i}$$

ν∈(O,1],ξi≥0

where *w* is the weights of the decision boundary, *b* is the intercept of the boundary and ξi is nonzero slack variable which allow the procedure to incur in errors. The parameter nu (ν) characterizes the solution as a) it sets an upper bound on the fraction of outliers (training examples regarded out-of-class) and, b) it is a lower bound on the number of training examples used as Support Vectors.

OCSVM can be effective for imbalanced classification where there are very few examples of the minority class. We report the hyper-parameters search space for binary SVM and OCSVM in S1 File.

We report the mean F1 and recall score as evaluation metric from each validation fold of K = 10-fold cross validation repeated 3 times. We did not use the ROC AUC score following the article by Takaya Saito [30]. The article highlights that highly imbalanced datasets tend to drag down the false positive rate due to an abundance of true negatives. Precision-Recall curves are more appropriate for imbalanced datasets. Thus, we chose to use the F1 score which represents the harmonic mean of precision and recall.

We choose K = 10 since the small values of K have high biases according to Kuhn and Johnson [31]. As K gets larger, the difference in size between the training set and the resampling subset becomes smaller. Moreover, leave-one-out cross-validation is more computationally expensive and Molinaro et al. [32] found that leave-one-out and k = 10-fold cross-validation yielded similar results. They also indicate that repeating k-fold cross-validation can be effectively used to increase the precision of the estimation and still maintain a small bias.

## Results and discussion

### Data preprocessing

**Fig 4:**
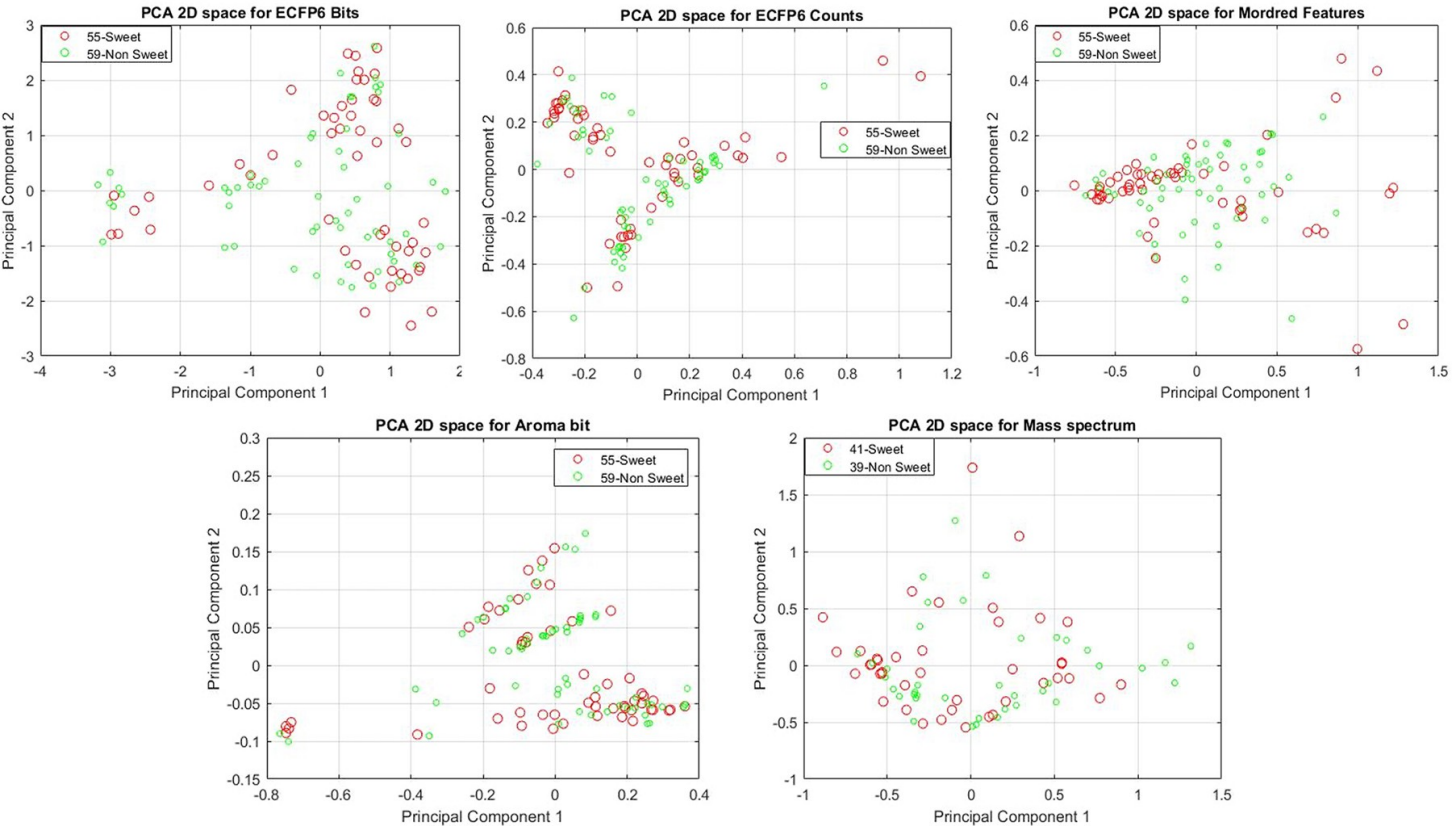
Principal component Analysis (PCA) for each feature set for sweet vs. non-sweet odor samples. For ECFP6 bit & count, Mordred and Aroma Bit features, we projected 114 aroma molecules in PCA-2D space. For the mass spectrum representations, we project the subset of 80 aroma molecules that were available in our dataset.

All features were preprocessed before feeding them to the machine learning model. We normalized the Mordred, Aroma Bit & mass spectrum representations to scale them into the range [0,1]. After that, we used principal component analysis (PCA) to remove the highly correlated components in the feature vectors. We keep 95% of total variance for each feature in this study. We listed the original dimension of each representation and their reduced dimension after PCA in Table 1. Fig 4 also depicts all those features in two dimensional PCA space for sweet & non-sweet odor descriptors. 35 principal components (PCs) explained 95% of the total variance for Mordred features. Bit and count-based molecular fingerprints need respectively 83 and 70 PCs for describing that percentage of variance. As the correlation among different channels of the Aroma Bit features is very high (Fig S2 in S1 File), we found that only 5 PCs are enough for modeling the odor prediction tasks. As explained in the previous section, the mass spectrum results are obtained on a subset of 80 aroma molecules given that the remaining samples were not available in our dataset. We found a reduced dimension of 39 PCs.

**Table 1:** Original and reduced dimension for each feature used in this study.

Features	Original Dimension	Reduced Dimension (95% variance)
ECFP6 bFP	1024	83
ECFP6 cFP	1024	70
Mordred	1324	35
Mass Spectrum	212	39
Aroma Bit	35	5

### Fine-grained odor descriptors prediction

Odor descriptor prediction is used for quality checking of products in the perfume, food, or cosmetic industries. A predictor can save time and cost as compared to manual assessments by human testers. In a first experiment, we use 114 aroma molecules with all the baseline features except for mass spectrum data (because not all the mass spectra are available from the NIST database) in a task to predict the presence or absence of 93 odor descriptors. We model this multi-label classification task using 93 corresponding binary classifiers. Traditional SVMs work well for the top odor descriptors where the ratio of negative to positive samples is not large. For other descriptors, as the number of target samples decreases, the prediction performance degrades due to imbalance between positive and negative examples. Thus, we utilize one-class SVM, as explained in our Materials section. The training procedure of one-class SVMs is the same as binary SVMs with the exception that the binary version uses both positive and negative samples during training and the one-class version uses only the majority class.

**Fig 5:**
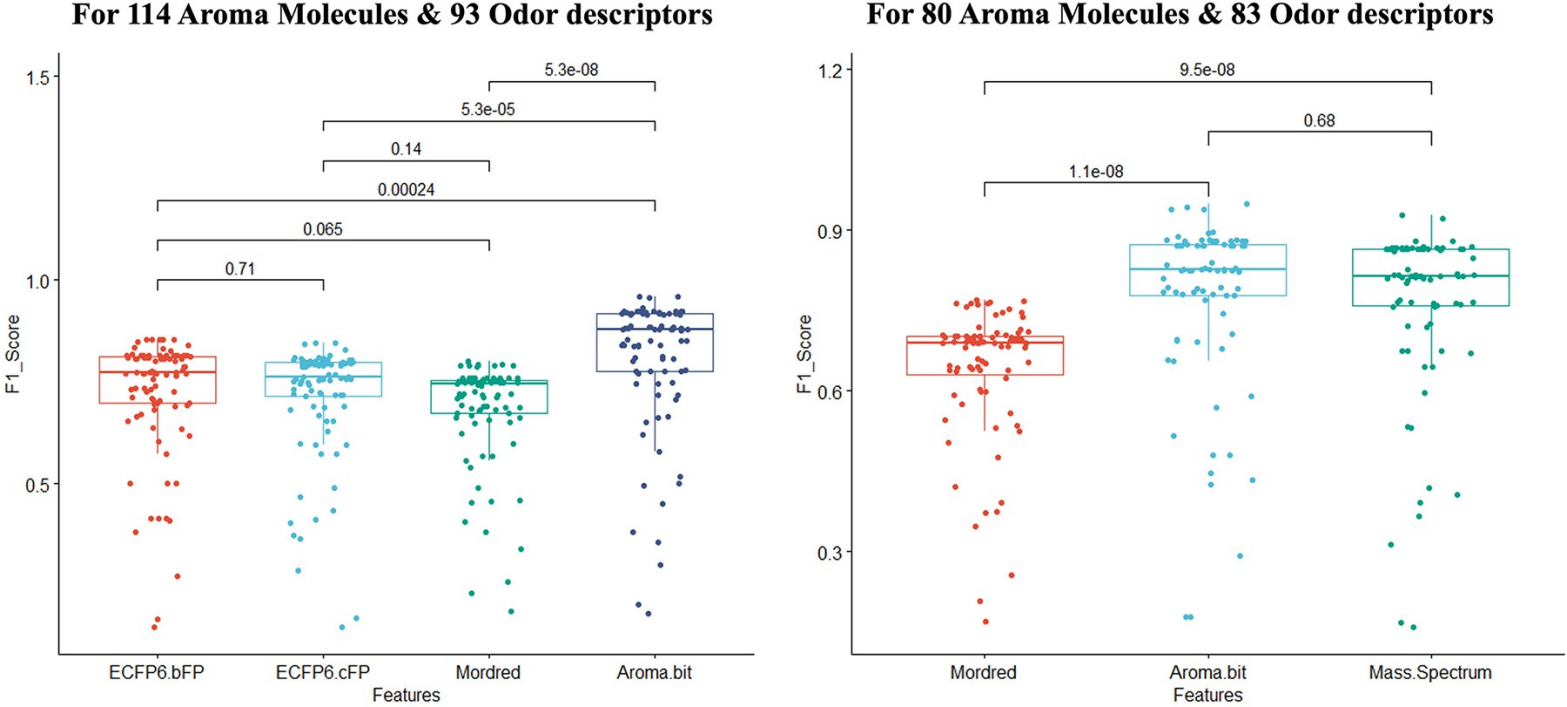
Fine grained odor descriptors prediction using One-class Support Vector Machine. (Left) figure depicts the statistical comparison of the pre-diction performance (F1 score) for 93 odor descriptors using 4 different features. (Right) figure shows the comparison among Mordred, Aroma Bit and mass spectrum features for 83 odor descriptors of 80 aroma molecules. Values indicate the p-value for the pairwise comparison between two features.

Statistical tests (paired t-test) across the 93 odor descriptors between each pair of features show no statistically significant difference between the results obtained with ECFP6 bit & count features and between ECFP6 count & Mordred features. As the prediction performance of these physicochemical features were almost the same, we used only Mordred features in the following experiments. However, the Aroma Bit features significantly outperformed other features. Fig 5 (left) shows the statistical test results with their p-value for each combination of the features where each data point is the F1 score of each of the 93 odor descriptors.

Next, we conducted the same experiment with the 80 aroma molecules for which we found mass spectrum representations. Only 83 odor descriptors are present in this subset of data. Again, we compared the predictive performance of each odor descriptor predictor based on F1 evaluation metric. The predictors based on Aroma Bit features significantly outperformed the ones based on Mordred features (as we found for 93 odor descriptors) but there was no statistically significant difference between the mass spectrum and Aroma Bit features with a p-value of 0.68 (largely far above the 5% significance level), see also Fig 5 (right). We reported the mean F1 & recall scores for both experiment in S2 and S3 Data.

These results are encouraging for two reasons. First, the two best-performing sets of features, the Aroma Bit (QCM based sensor) and mass spectrum features, can also be obtained for chemical mixtures. Future research should explore if their predictive performance can be generalized on mixtures, a great target for machine olfaction. Secondly, it is easy to use QCM-based sensors. Compared to these sensors, mass spectrometry sensing devices are much more expensive and bulkier. We hope that our results will encourage the apparition of new datasets based on e-noses for conducting more experiments. However, such sensors commonly present sensor drift after a certain period, which could hinder the creation of large-scale QCM-based data.

The physicochemical properties described previously (bFP, cFP, Mordred features) cannot be a good choice for chemical mixtures. Let us imagine processing a chemical mixture made of two mono-molecular subcomponents. If we added up the chemical properties of the subcomponents, the summation could represent a completely different element than the chemical mixture in terms of boiling point, vapor pressure, etc. Thus, as we conduct our experiments on mono-molecular odorants, it is worth noting that only the mass spectrum and Aroma Bit sensing data can potentially generalize on mixtures.

### Odor descriptor group prediction

**Fig 6:**
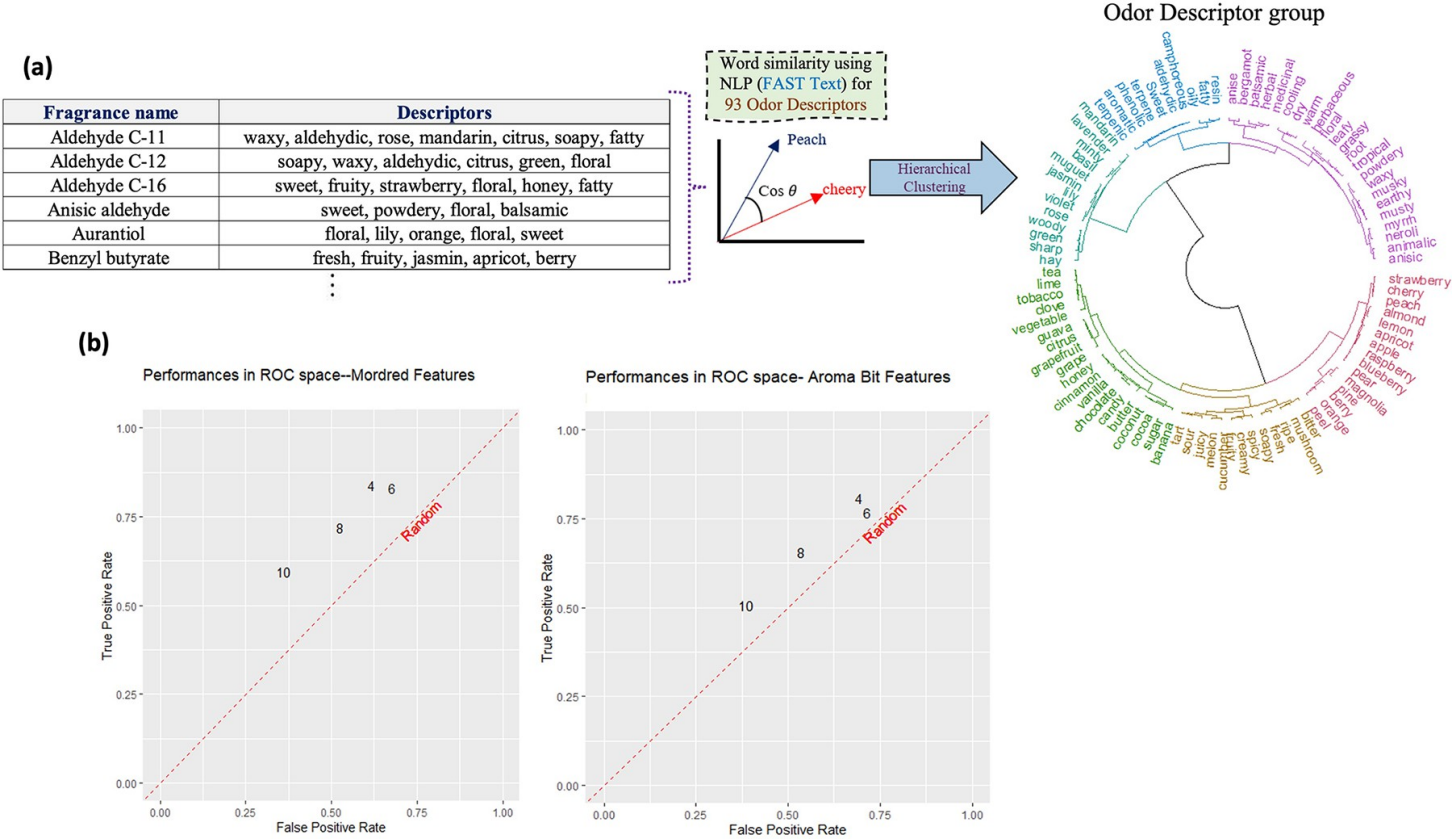
(a) Clustering the 93 odor descriptors in groups using natural language processing (Fast-Text). Results for 6 odor clusters created using hierarchical clustering on the word-similarity matrix. We used these odor descriptor groups as target vectors for new group-based predictive models. If a sample has one of the descriptors which is in a cluster i, then this sample is considered as a positive example of that cluster i.; (b) Prediction performance in ROC space for Mordred features (left side) & Aroma Bit features (right side).

Although aroma molecules can be described with several odor descriptors, these descriptors are often correlated with one another. For example, one can argue that the ‘raspberry’ and ‘blueberry’ labels are correlated. As such, in an alternative task, instead of predicting each specific odor descriptor, we can predict the similar kind of odor impressions together. This is a two-step process. First, we cluster odor descriptors in similar groups. The procedure is a downstream task where pre-trained word vectors were used for calculating the cosine similarity among 93 odor descriptors. Then, we predict the presence or absence of a group of odor descriptors for a desired aroma molecule. Our odor descriptor clustering strategy used pretrained word vectors on the English Wikipedia corpus using natural language processing (Fast-Text) to calculate the similarity among odor descriptors as explained in the article [22]. English pre-trained word vector (300 dimensions) was downloaded (6.59 GB) and there are 2519370 tokens available. After that, pair-wise cosine similarities between odor descriptors are calculated using their word vectors. Finally, hierarchical clustering was carried out based on the cosine similarity matrix. Fig 6(A) illustrates the procedure for building 6 odor descriptors cluster. We conducted our experiments with four different numbers of clusters; their respective descriptors group are reported in (Fig S1 in S1 File).

The odor wheel in Fig 6(A) pictures the hierarchical clustering and qualitatively describes the association of similar kinds of smell impressions. For example, “candy” is adjacent to “chocolate” and “vanilla” on the wheel. This signals to perfumers and chemists that when someone describes a candy flavor, it could be similar to a chocolate or vanilla flavor according to linguistic clustering.

After preparing the odor descriptor groups, we used them as an output vector for the binary SVM classifier. If at least one descriptor in an odor group is present in a sample, then the odor group is considered positive for that sample. Otherwise, it is considered negative. The number of clusters is a hyperparameter that strongly affects the distribution of samples and the performance of the predictive model. Fig S3 in S1 File shows the distribution of the 114 aroma molecules in the different cluster settings. For example, with 4 odor descriptor groups, there are 107 positive samples for cluster index 1. This means that most of the descriptors belong to one huge cluster while cluster index 2 include very small number of descriptors.

We used only Mordred features and Aroma Bit features for this task because the prediction performance of molecular fingerprints and the performance of the Mordred features were similar in the previous task. As expected, we find different prediction performance depending on the number of groups in the clustering step. The best performing results are obtained with 6 clusters, where we found F1 scores of 0.70 and 0.65 for Mordred features and Aroma Bit, respectively. True positive rate is 0.83 & 0.76 for Mordred features and Aroma Bit, respectively. With other numbers of clusters, the performance deteriorated. This is pictured in Fig 6(B). Among all different cluster configurations, we didn’t find a statistically significant difference between the performance obtained with the two feature sets.

### Qualitative analysis of the feature clustering

**Fig 7:**
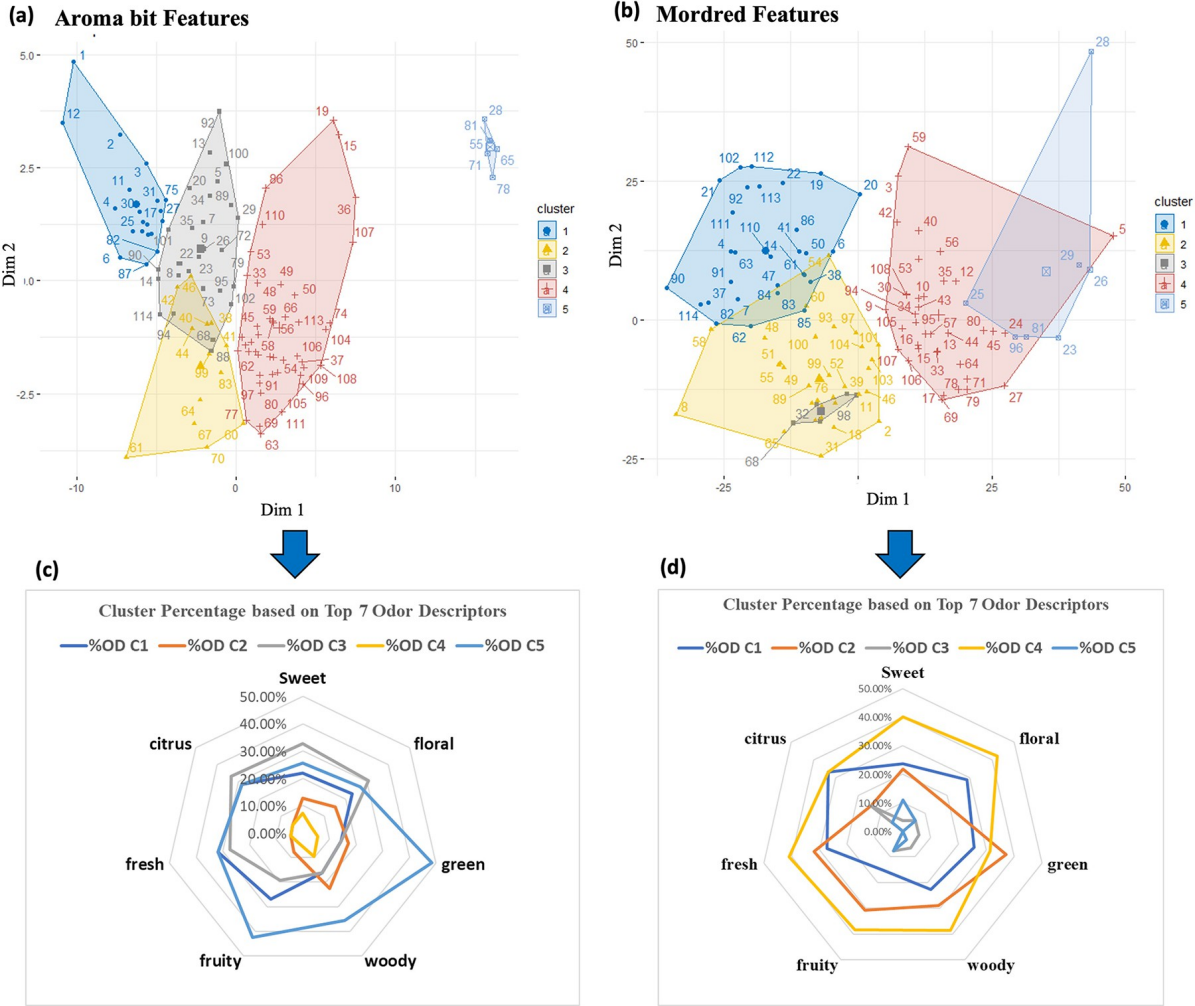
Hierarchical clustering of the Principal Components of aroma molecules based on (a) Aroma Bit and (b) Mordred features. In the top figures (a) and (b), each number indicates the index of the molecule (see S1 Data). We analyzed the distribution of the percentage of the 7 most frequent odor descriptors across the 114 aroma molecules in (c) Aroma Bit & (d) Mordred features. For example, 32.73% sweet in cluster 3 using aroma bit features would mean that 32.73% of all sweet samples are gathered in the cluster 3.

In this final comparative task, we performed the analysis of the cluster composition of the 114 aroma molecules. Hierarchical clustering was performed on the PC components of both aroma-bit features (5 PCs >90% variance) and Mordred features (35 PCs> 90% variance) and is illustrated in Fig 7(A) and 7(B) respectively. This is not to be confused with the clustering of odor descriptor groups in the previous task. Here, we cluster the feature space of the predictors, not the output space of the labels. An optimal number of 5 clusters was selected based on the “Elbow” curve method (representation of intra-cluster variability as a function of the number of clusters), as reported in Fig S4 in S1 File.

We denote the percentage of an odor descriptor in a cluster as the ratio between the number of samples inside the cluster that present this odor descriptor over the total number of samples that present this odor descriptor. For example, a percentage of 80% ‘sweet’ in cluster 1 would mean that 80% of the ‘sweet’ samples are gathered in cluster 1. Fig 7 depicts the radar charts of the distribution of odor descriptor percentage values for the 7 most frequent odor descriptors across the 114 aroma molecules. There is a total of 55 ‘sweet’ and 52 ‘floral’ samples among the 114 aroma molecules in our original database. We found that 32. 73% sweet and 30.77% floral odor descriptors are in the 3^rd^ cluster for Aroma Bit features in Fig 7(C), and 40% sweet and 42% floral are in the 4^th^ cluster for Mordred features in Fig 7(D). However, notice that all the percentages of the descriptors for sweet, floral, woody, fruity, fresh, and citrus, are high in the 4^th^ cluster for the Mordred features. It is more difficult for a predictive model to differentiate the descriptors inside of this cluster. Also, notice that the observation for the green, woody, and fruity odor descriptors appear in a common cluster (5) with the Aroma Bit features. Qualitatively, the Aroma Bit features seem to present better clustered profiles compared to the Mordred features based on the percentage of the odor descriptors. This was also demonstrated by the predictive models in our first task, as we obtained true positive rates of 83% and 60% for sweet and floral odor descriptors respectively using the Aroma Bit features, compared to 49% and 36% for the Mordred features.

## Conclusion

We experimented with different featurization methods of olfactory stimuli for predicting not only the specific odor impression but also the odor descriptor group of aroma molecules. Our aim was to compare the predictive performance in terms of different sensing and feature data. We found that the QCM-based Aroma Bit sensor features, and the mass spectrum features gave similar prediction performance of odor impression. Also, they both significantly outperformed molecular descriptor data (bit-based path descriptors fingerprint, count-based Morgan fingerprints, Mordred features) in the same task. This result is encouraging as 1) contrarily to molecular descriptor data, QCM-type and mass spectrum features can be used with chemical mixtures as well, and 2) QCM-based sensors are relatively inexpensive and easy to use. We also analyzed the cluster composition with Aroma Bit and Mordred features and found that the Aroma Bit clusters gathered less olfactory notes in the same cluster as compared to the Mordred features, which makes it easier to distinguish between target and non-target odor impressions.

We provide the dataset of QCM-type features we created for our aroma molecules and encourage researchers to conduct additional comparative experiments. We hope these findings will help researchers experiment with new types of features for olfactory representation and build new datasets of odor samples. Importantly, we hope to see datasets investigating the sensory attributes of chemical mixtures in the food, perfume, or other related industries, as this would be a new milestone for olfactory research.

## Supporting information

S1 Data Contains the aroma molecules with their CAS number, odor descriptors, aroma bit features (average of 3 measurements) and 80 aroma molecules with mass spectrum. (XLSX)

S2 Data Reports the mean F1 & recall scores for 93 & 83 Odor descriptors respectively. (XLSX)

S3 Data Reports the mean F1 & recall scores for 93 & 83 Odor descriptors respectively. (XLSX)

S1 File Reports the hyper-parameters search space, odor descriptor group, distribution of clusters among 114 aroma molecules and the optimal number of clusters suing Elbow curve method. (PDF)
