# Supplementary material for: Comparing molecular representations, e-nose signals, and other featurization, for learning to smell aroma molecules
Source: PLoS One. 2023 Aug 11;18(8):e0289881. doi: 10.1371/journal.pone.0289881 (PMC10420360; doi:10.1371/journal.pone.0289881)

Comparing molecular representations, e-nose signals, and other featurization, for learning to smell aroma molecules

Tanoy Debnath*, Samy Badreddine, Priyadarshini Kumari, Michael Spranger

*Tanoy Debnath- Sony AI, Tokyo, Japan

https://orcid.org/0000-0002-2624-8923

E-mail: kuettanoydebnath@gmail.com

Table S1: Hyperparameter search Space

Algorithm	Parameters Space
SVM	'C': [0.01, 0.1, 1, 10, 100],<br>'gamma': [100,10,1,1e-1,1e-2, 1e3],<br>'kernel': ['linear','rbf']
One Class SVM	'kernel': ['linear','rbf'],<br>'gamma': ['scale', 'auto'],<br>'nu': [100,10,1,1e-1,1e-2, 1e-3]

Fig S1: Odor Descriptor Group Clustering based on Natural Language processing-based word-similarity among 93 odor descriptors. Here we illustrated three odor descriptors group (6 clusters OD group depicted in Fig 6 (a)-main manuscript).

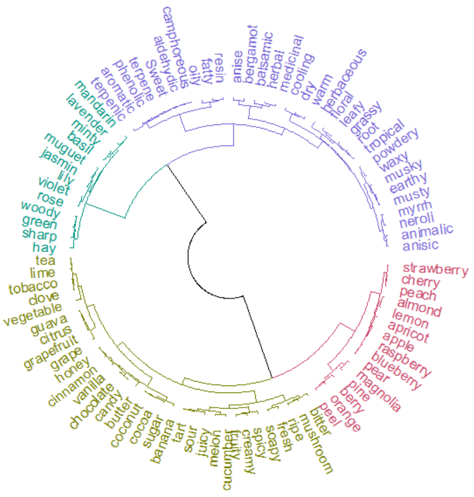

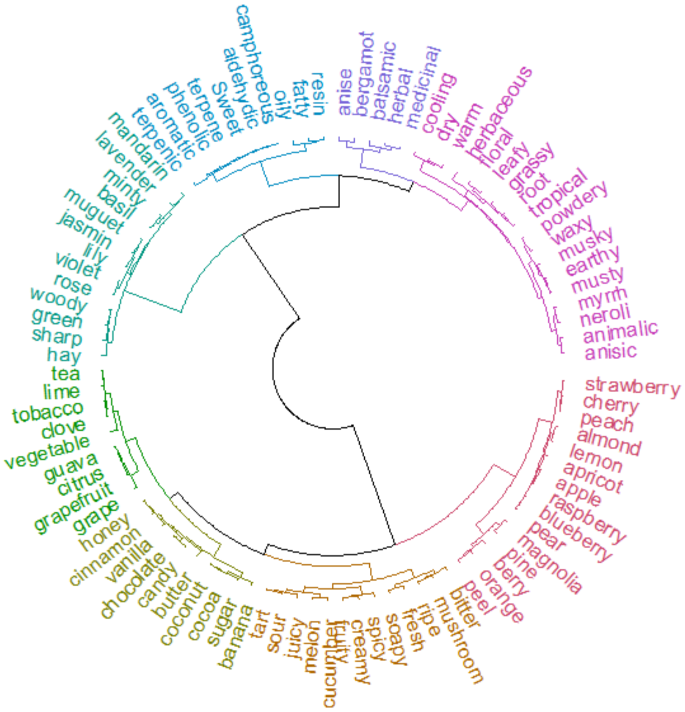
resin
fatty
oily
camphoreous
aldehydic
Sweet
terpene
phenolic
aromatic
terpenic
mandarin
lavender
minty
basil
muguet
jasmin
lily
violet
rose
woody
green
sharp
hay
tea
lime
tobacco
clove
vegetable
guava
citrus
grapefruit
grape
honey
cinnamon
vanilla
chocolate
candy
butter
coconut
cocoa
sugar
banana
tart
sour
juicy
melon
cucumber
fruity
creamy
spicy
soapy
fresh
ripe
mushroom
bitter
peel
orange
berry
pine
magnolia
pear
blueberry
raspberry
apple
apricot
lemon
almond
peach
cherry
strawberry
anisic
animalic
neroli
myrrh
musty
earthy
musky
waxy
powdery
tropical
root
grassy
leafy
floral
herbaceous
warm
dry
cooling
medicinal
herbal
balsamic
bergamot
anise

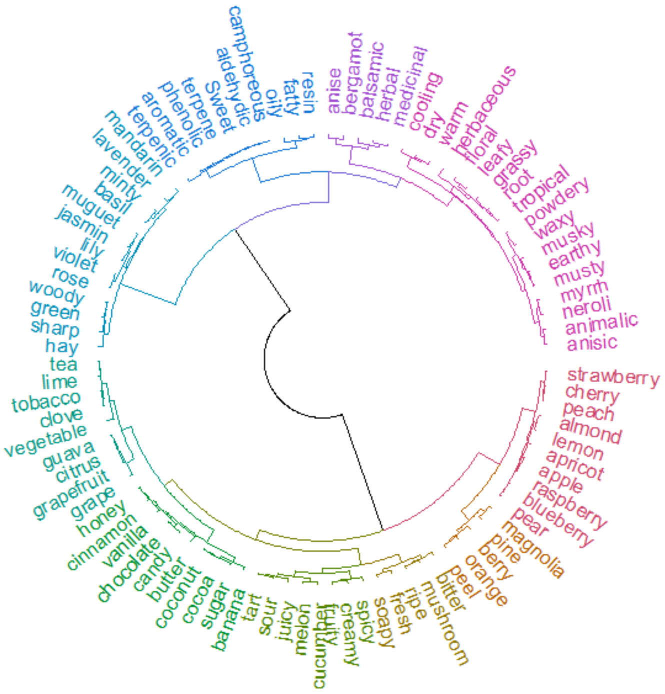
resin
fatty
oily
camphoreous
aldehydic
Sweet
terpene
phenolic
aromatic
terpenic
mandarin
lavender
minty
basil
muguet
jasmin
lily
violet
rose
woody
green
sharp
hay
tea
lime
tobacco
clove
vegetable
guava
citrus
grapefruit
grape
honey
cinnamon
vanilla
chocolate
candy
butter
coconut
cocoa
sugar
banana
tart
sour
juicy
melon
cucumber
fruity
creamy
spicy
soapy
fresh
ripe
mushroom
bitter
peel
orange
berry
pine
magnolia
pear
blueberry
raspberry
apple
apricot
lemon
almond
peach
cherry
strawberry
anisic
animalic
neroli
myrrh
musty
earthy
musky
waxy
powdery
tropical
root
grassy
leafy
floral
herbaceous
warm
dry
cooling
medicinal
herbal
balsamic
bergamot
anise

Fig S2: correlation among different channels of the Aroma Bit features

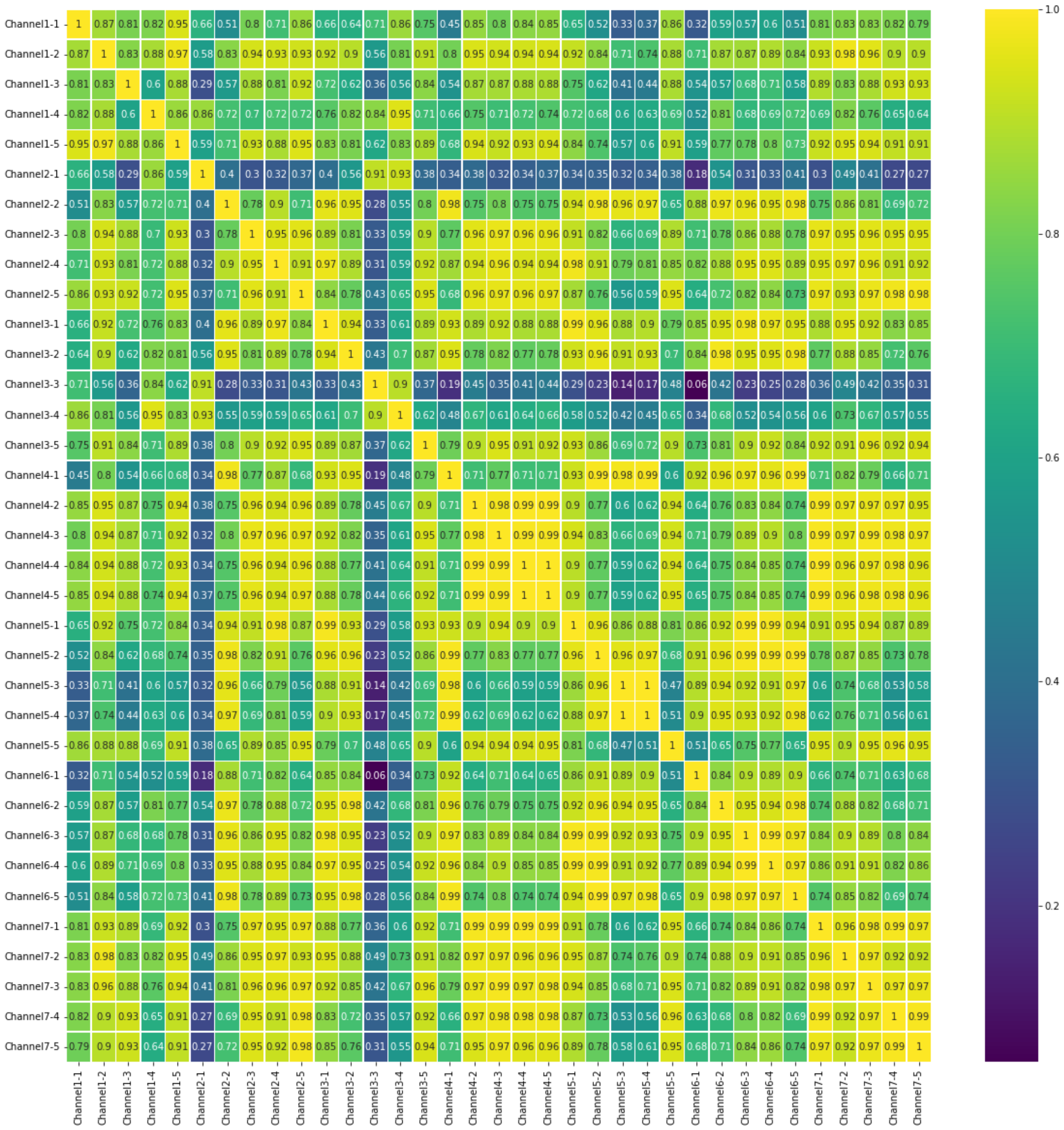

Fig S3: Odor Descriptor Group Prediction - Distribution of clusters among 114 aroma molecules.

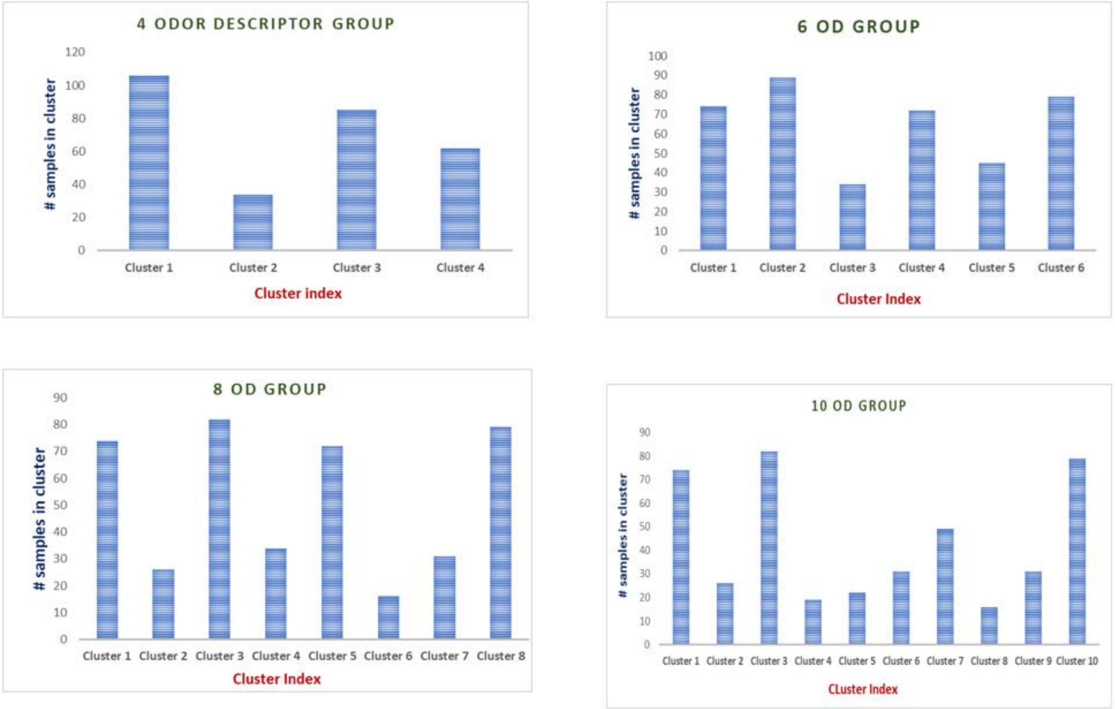

Distribution of 114 aroma molecules with respect to odor descriptor group

Fig S4: Optimal number of clusters (5) was selected based on the "Elbow" curve method (Representation of intra-cluster variability as a function of the number of clusters)

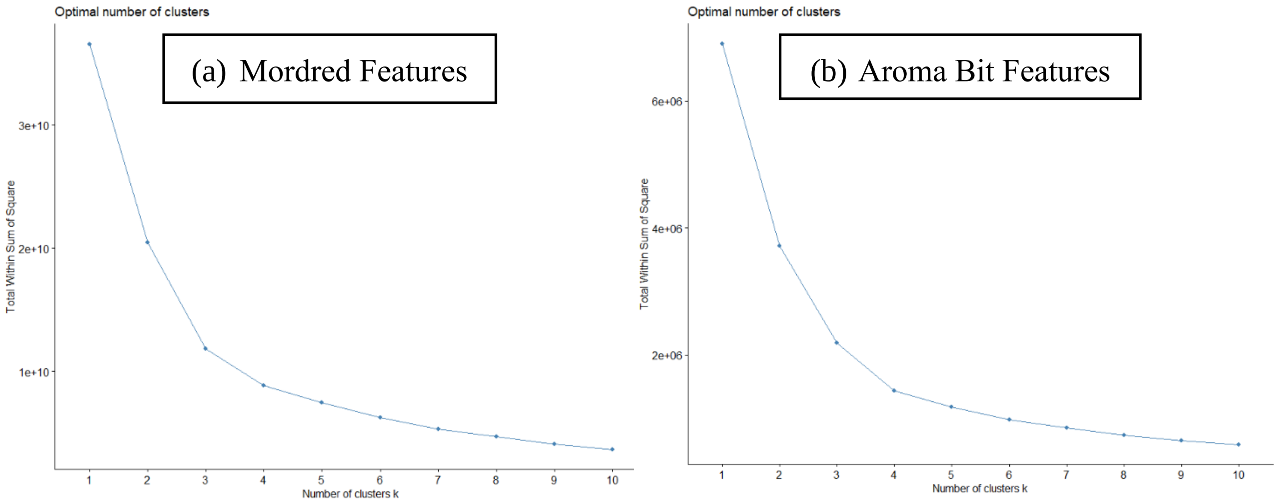

Supplement: S1 File — (PDF) [file pone.0289881.s004.pdf]
